# The Responses of Sucrose Metabolism and Carbon Translocation in Tomato Seedlings under Different Light Spectra

**DOI:** 10.3390/ijms242015054

**Published:** 2023-10-10

**Authors:** Xiaoxu Zhan, Qichang Yang, Sen Wang, Yu Wang, Xiaoxue Fan, Zhonghua Bian

**Affiliations:** 1 Photobiology Research Center, Institute of Urban Agriculture, Chinese Academy of Agricultural Sciences, Chengdu 610000, Chinayangqichang@caas.cn (Q.Y.); wangyu05@caas.cn (Y.W.); 2 Institute of Agricultural Information, Key Laboratory of Intelligent Agricultural Technology (Changjiang Delta), Institute of Agricultural Information, Ministry of Agriculture and Rural Affairs, Jiangsu Academy of Agricultural Sciences, Nanjing 210014, China

**Keywords:** light quality, sucrose-metabolizing enzymes, carbon translocation, gene expression, tomato

## Abstract

Light plays a dominant role in the biosynthesis and accumulation of photosynthetic products. However, the metabolism and translocation of photosynthetic products in plants under different light spectra remain elusive. In this study, tomato (*Solanum lycopersicum* L.) seedlings were treated with different light spectra delivered by light-emitting diodes (LEDs) with the same photosynthetic photon flux density at 300 μmol m^−2^ s^−1^, including monochromatic red (660 nm, R), blue (450 nm, B), sun-like white (W, 380–780 nm), or a combination of R and B lights (R:B = 1:1, RB). Compared with W, the biomass distribution ratio for leaves under R, B, and RB decreased by 5.01–9.53%, while the ratio for stems and roots increased by 3.71–6.92% and 0.14–2.81%, respectively. The photosynthetic carbon distribution expressed as ^13^C enrichment was higher in stems and roots under RB and R, while B led to more ^13^C transported from leaves and enriched in stems when compared with W. Meanwhile, RB led to significant increases in the activities of phosphate synthase (SPS), sucrose synthase (SS), vacuolar acid invertase (VI), and neutral invertase (NI). The R was more efficient in increasing the activity of SPS and SS, while B was more effective in promoting the activity of VI and NI. The transcript levels of *SPS*, *SS3*, *NI6*, and *VI* were upregulated under R, B, and RB. However, the transcript patterns of *SPS*, *SS3*, *NI6*, and *VI* were not consistent with the changes in their encoded enzymes, especially the transcript patterns of *SPS* and *SS3*. Our study suggests that the red- and blue-light-induced long-distance and short-distance transport of photosynthetic products in plants, respectively, might result from different regulation of sucrose-metabolizing enzymes from transcriptional and post-transcriptional levels.

## 1. Introduction

Light is the main energy source for driving plant photosynthesis, and it also acts as an important environmental signal in the regulation of plant growth and development [1,2]. Artificial lighting is one of the most important environmental regulation strategies in greenhouse production. Light-emitting diodes (LEDs) are considered an innovative light source for the production of horticultural crops [3] and are also widely used in plant photobiology investigation [4,5] because of their high photoelectric conversion efficiency, monochromatic light, and flexible and accurate light spectral combination. It is well-known that the effects of light on photosynthesis, growth, and development can be categorized into light intensity, photoperiod (or light duration), and light quality [6,7]. Since higher plant leaves have strong absorption at the wavelengths of blue (400–500 nm) and red (600–700 nm) light, red and blue light are widely studied and a combination of red and blue is more efficient in regulating photosynthesis and morphogenesis, including biomass accumulation and photosynthetic-related gene expression [8].

Tomato is the largest horticultural crop grown in greenhouses worldwide, and it is also a light-loving crop [9]. To achieve high yield and quality, a combined red and blue LED light is commonly used in tomato production in a greenhouse, especially during off-season production [10]. Sucrose is the main end product of photosynthetic substance and the principal form in which carbon is transported from leaves to other sink organs through the phloem to supply carbon and energy for the growth and development of plants [8,11]. In plants, the synthesis, accumulation, and transportation of sucrose depend on the concerted action of sucrose-metabolizing enzymes, including sucrose phosphate synthase (SPS; EC 2.3.1.14), sucrose synthase (SS; EC2.4.1.13), vacuolar/soluble acid invertase (VI, EC 3.2.1.26), and neutral invertase (NI, EC 3.2.1.27) [12]. In tomato plants, SS and SPS mainly catalyze the synthesis of glucose and fructose to sucrose, while VI and NI catalyze the hydrolysis of sucrose to glucose and fructose [13].

In some plants, SS activity is the main route for the entry of sucrose into cellular metabolism. A reduction in SS activity led to a decrease in the availability of assimilate for storage and normal growth [14]. For instance, inhibition of SS activity in tomatoes decreased the fruit setting and sucrose unloading capacity [15].

In plants, the changes in sucrose-metabolizing enzyme activity are subjected to transcription, translation, and post-translational modification mechanisms of genes encoding these enzymes. Qin, et al. [16] identified six *SS* genes and four *SPS* genes in the tomato genome. Among those genes, *SPS* and *SS3* were proved more sensitive to environmental factors, especially at special growth periods [17]. In addition, eleven invertase genes were identified in tomato plants, of which five are predicted to encode cell wall invertase protein CWI (*LIN5-9*), one encodes a vacuolar invertase VI (*VI*) [16], and five encode neutral invertase NI (*NI1-6*) [18]. Compared with CWI, VI plays vital biological functions related to hydrolyzing sucrose, and sucrose metabolism for growth and development [19]. Furthermore, *NI6* mRNA was proved to be present in all organs of tomato plants, encoding the cytosolic invertase NI, which shows a broad range of functions, including vegetative growth, flowering, fruit set, and yield in tomatoes [18].

A previous study has proved that sucrose metabolism in tomato seedling leaves was affected by light conditions via enhancing the activity of SS and SPS [8]. The related gene expressions of the sucrose-metabolizing enzymes were affected by external environmental cues, such as temperature, light, and pathogen infection [11,20]. However, the spatial transcript patterns of sucrose-metabolizing enzymes in different organs of plants remain unclear, as well as whether the changes in these related gene expression levels would result in similar changes in enzyme activity in plants under different light spectra.

Carbon translocation is an integral component of the growth and yield-determining processes in higher plants. In plants, carbon translocation is also affected by light conditions, including light quality (light spectra), light intensity, and photoperiod [21,22]. Compared with light intensity and photoperiod, the effects of light quality on substance synthesis, metabolism, and translocation were more complex [3,23]. However, our knowledge of carbon fixation and translocation concerning light quality is currently quite incomplete. As an important model for plant research and also as a worldwide grown crop in the greenhouse, it is important to know the intricate effect of light quality on leaf-fixed carbon translocation in tomato plants.

This study aims to further investigate the responses of sucrose metabolism and carbon translocation in plants under different light spectra from the physiological and molecular levels. In this study, a combination of sucrose-metabolizing enzyme assays and transcript expression analysis was used to investigate the potential relationship between the related gene expression of sucrose-metabolizing enzyme and changes in enzyme activities under different light spectra. In addition, we used a steady-state ^13^CO_2_ labeling technique to verify the photosynthetic carbon translocation in tomato seedlings exposed to LEDs with different spectral compositions. The results of this present study not only could provide guidance on high-quality tomato production in greenhouses via artificial lighting but could contribute to a better understanding of the regulation of light quality on crop growth and development from the levels of carbohydrate synthesis, accumulation, and translocation.

## 2. Results

### 2.1. Effects of Light Spectra on the Growth of Tomato Seedlings

The plant growth and biomass distribution of tomato seedlings were significantly affected by light spectra compositions (Table 1). Compared with white light control (W), monochromatic red and blue light led to decreases in leaf areas, but a combination of red (R) and blue (B) light promoted tomato leaf area increase, as shown by the significantly higher leaf area of RB than that of W. The dry weight (DW) under RB was significantly higher than W, but R and B exposure led to decreases in DW. The dry mass distribution in plants under R, B, and RB showed opposite trends of plants under W. Compared with W, the biomass distribution ratio for leaves under R, B, and RB decreased by 5.01–9.53%, while the ratio for stems and roots increased by 3.71–6.92% and 0.14–2.81%, respectively. The dry mass partitioning to leaves was lowest, but dry mass partitioning to stems and roots under RB was highest among different light treatments. The significant difference between R and B was only observed in the dry mass partitioning to stems.

### 2.2. The Responses of Photosynthetic Performance of Tomato Seedlings under Different Light Spectra

**Figure 1:**
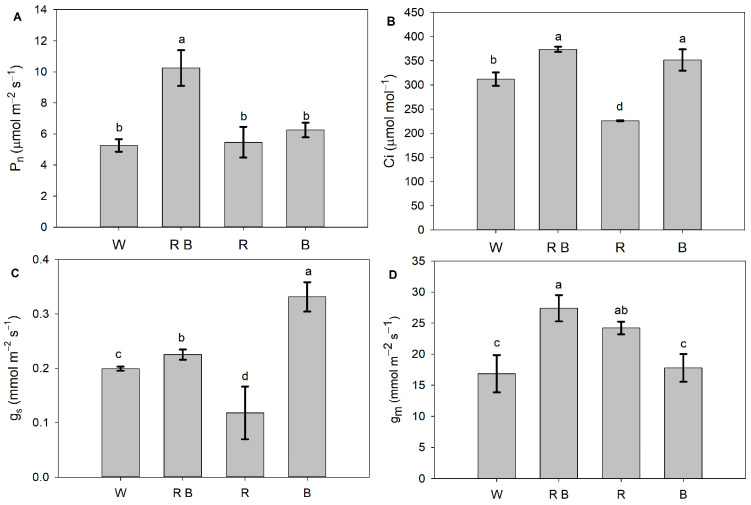
The photosynthetic responses and mesophyll conductance of tomato seedlings exposed to different LED lights. (**A**) The net photosynthetic rate (P_n_); (**B**) intercellular CO_2_ concentration; (**C**) stomatal conductance (g_s_); (**D**) mesophyll conductance (g_m_). W, RB, R, and B represent white light treatments, combined red and blue light treatments, monochromatic red light treatments, and monochromatic blue light treatments, respectively. Different lowercase letters indicate significant differences at the 0.05 level. Values represent the average of three biological replicates ± SD.

A combination of red and blue light significantly enhanced the net photosynthetic rate (P_n_), as shown by the higher P_n_ under RB than that under other light treatments. However, the P_n_ of R and B were comparable to that of W (Figure 1A). Compared with W, RB and B enhanced intercellular CO_2_ concentration (C_i_), while R led to a marked decrease in Ci (Figure 1B). The stomatal conduction (g_s_) value was highest under B, followed by RB, W, and then R (Figure 1C). The values of mesophyll conductance (g_m_) under RB and R were higher than that under W, but the g_m_ under B was comparable to that under W (Figure 1D).

### 2.3. Carbohydrate Contents in Plants under Different Light Spectra

The contents and distribution of glucose, sucrose, and starch in tomato plants were significantly affected by light spectra (Table 2). In leaves, the contents of glucose, sucrose, and starch were highest under RB, while the lowest contents of these parameters (except glucose) were observed under B. In studied foliar carbohydrates, the contents of glucose and sucrose under R were significantly increased compared to those under W. In stems, the highest contents of glucose, sucrose, and starch were still observed under RB. Compared with W, R enhanced sucrose accumulation in stems, but R and B led to starch decreases in stems. RB enhanced glucose accumulation in tomato roots when compared with the other three light treatments. In contrast, R, B, and RB led to decreases in sucrose and starch in roots, with the lowest values of sucrose and starch under B.

### 2.4. Organic Carbon and Isotope Composition of Carbon in Plants under Different Light Spectra

To investigate the effects of light spectra on photosynthesis-fixed carbon accumulation and translocation, we carried out a pulse labeling experiment using stable carbon isotopes. Under different light spectral treatments, the significant difference in organic carbon was only observed in the roots of tomato seedlings. Compared with white light control, red, blue, and a mixture of red and blue led to increases in root organic carbon, as shown by the highest organic carbon under RB, followed by B and R (Table 3). When compared with W, the ratio of ^13^C/^12^C (δ^13^C) was decreased in tomato leaves and roots but was increased in stems under B. Similarly, RB led to a decrease in δ^13^C in roots and an increase in stems. Interestingly, monochromatic red light exposure led to δ^13^C increase in different organs of plants, as shown by the higher values of δ^13^C in leaves, stems, and roots of plants under R (Table 3).

### 2.5. The Distribution of Photosynthetic Products under Different Light Spectra

**Figure 2:**
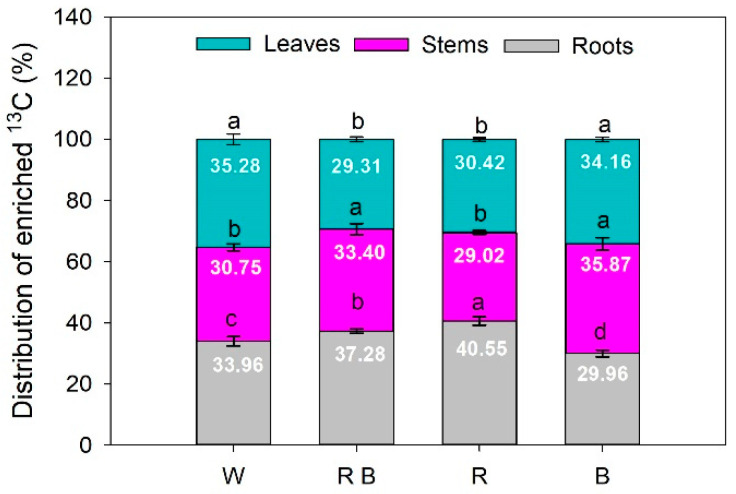
The ^13^C distribution in tomato seedlings exposed to different light treatments. Different lowercase letters indicate significant differences at the 0.05 level. Values represent the average of three biological replicates ± SE. W, RB, R, and B represent white light treatments, combined red and blue light treatments, monochromatic red light treatments, and monochromatic blue light treatments, respectively.

The distribution of photosynthetic products in plants was presented as the percentage of enriched ^13^C in different organs of tomato seedlings. The distribution of enriched ^13^C showed a similar change tendency as δ^13^C under different light treatments. Compared with white light control, red light promoted the transfer of photosynthetically fixed carbon from leaves and enriched in plant roots, as shown by the significantly higher percentage of ^13^C in roots, and a lower percentage in leaves under R than that under W (Figure 2). When compared with W, the percentage of enriched ^13^C in plant stems under B was increased, whilst B led to this parameter’s decrease in roots. This indicates that B was more efficient in promoting photosynthetically fixed carbon accumulation in plant stems. Interestingly, compared to W, RB significantly increased the percentage of ^13^C enriched in both stems and roots of plants, but caused a decrease in the percentage of ^13^C enriched in leaves (Figure 2). These results suggest red light and blue light contribute to the long-distance and short-term transportation of photosynthetic products in plants, respectively, and a combination of red and blue light has a complementary gain effect on this phenomenon.

### 2.6. Sucrose Metabolism Enzyme Activities and Related Gene Expression

**Figure 3:**
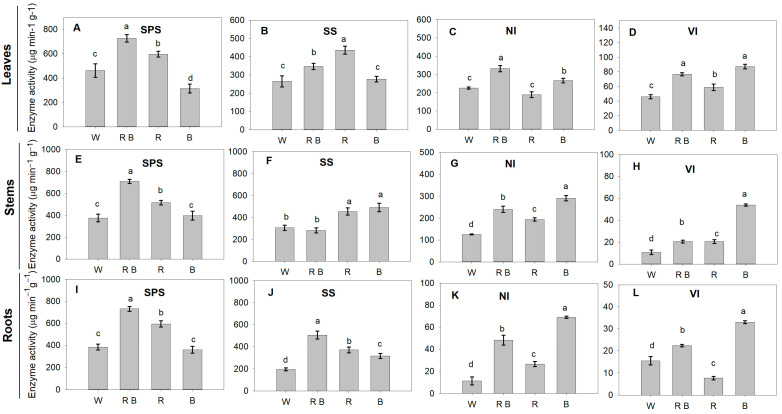
Effects of light spectra on the activities of enzymes involved in sucrose metabolism in leaves (**A**–**D**), stems (**E**–**H**), and roots (**I**–**L**) of tomato plants. SPS, sucrose phosphate synthase; SS, sucrose synthase; NI, neutral invertase; VI, vacuolar acid invertase. Different lowercase letters indicate significant differences at the 0.05 level. Values represent the average of three biological replicates ± SE.

The activities of these enzymes showed similar variations under different light treatments (Figure 3A–L). Compared with W, the activities of SPS in leaves, stems, and roots were significantly increased under RB and R, with the highest values observed in plants exposed to RB (Figure 3A,E,I). However, the SPS activity of leaves under B was significantly lower than that under W (Figure 3A). Concerning SS, the other important enzyme of sucrose biosynthesis, the highest activity in leaves was observed under R, followed by RB, B, and then W. However, no significant difference in leaf SS activity was observed between B and W (Figure 3B). In plant stems, the activities of SS under R and B were higher than that under W, whilst the activity did not differ significantly between R and B (Figure 3F). The activity of SS in roots under different treatments was kept in order: RB > R > B > W (Figure 3J).

The activities of NI in different parts of plants under RB and B were higher than those under W. However, compared with W, the significant increase in the activity of NI under R was only observed in stems and roots (Figure 3C,G,K). Similarly, the VI activities of RB and B were higher than that under W, with the highest value obtained under B in leaves, stems, and roots (Figure 3D,H). In addition, R led to increases in the activity of VI in plant leaves and stems, but caused a decrease in roots compared with W (Figure 3D,H,L).

**Figure 4:**
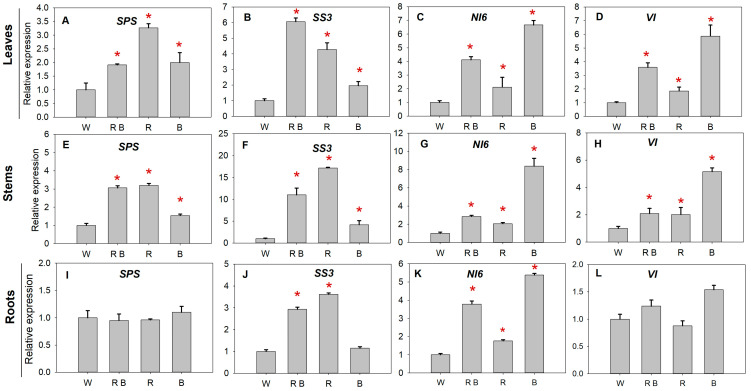
Effects of light spectra on the related gene expression involved in sucrose metabolism in leaves (**A**–**D**), stems (**E**–**H**), and roots (**I**–**L**) of tomato plants. *SPS*, sucrose phosphate synthase gene; *SS3*, sucrose synthase gene; *NI6*, neutral invertase gene; *VI*, vacuolar acid invertase gene. The “*” with red color above the columns indicates that the gene expression level was upregulated compared with white light control (W). W, RB, R, and B represent white light treatments, combined red and blue light treatments, monochromatic red light treatments, and monochromatic blue light treatments, respectively. Different lowercase letters indicate significant differences at the 0.05 level.

To reveal the effects of light spectra on the biosynthesis and metabolism of photosynthetic products from the molecular level, we investigated the transcript levels of some important genes related to sucrose biosynthesis and metabolism, including *SPS*, *SS3*, *NI6*, and *VI* (Figure 4A–L). Compared with W, the transcript levels of *SPS* under R, B, and RB were upregulated by 3.3, 2.0, and 1.9 times in tomato leaves (Figure 4A) and by 3.0, 3.2, and 1.6 times in stems, respectively (Figure 4E). The expression level of *SS3* under R, B, and RB was upregulated by 4.3, 2.0, and 6.1 times in leaves (Figure 4B) and by 17.2, 4.2, and 11.0 times in stems (Figure 4F) when compared with that under W. Furthermore, R and RB led to upregulation of *SS3* expression in roots, with a value of 3.0 and 2.9 times higher than that under W (Figure 4J).

The transcript pattern of *NI6* showed a similar change tendency as the SS activity under different light treatments. Compared with white light control, R, B, and RB induced the upregulation of *NI6* expression in plant leaves, stems, and roots with the higher transcript level obtained under B, followed by RB and R (Figure 4C,G,K). Compared with W, the expression levels of *VI* under R, B, and RB were upregulated by 1.9, 3.6, and 5.9 times in tomato leaves (Figure 4D) and by 2.0, 2.1, and 5.6 times in stems, respectively (Figure 4H). These results indicated that red light and blue light have different effects on the transcript levels of *SPS*, *SS3*, *NI6*, and *VI*. Red light was more efficient in upregulating *SPS* and *SS3* expression, while blue light easily induced the expression of *NI6* and *VI*. Furthermore, there was an interaction between red and blue light in terms of regulating the expression of those investigated genes (Figure 4).

**Figure 5:**
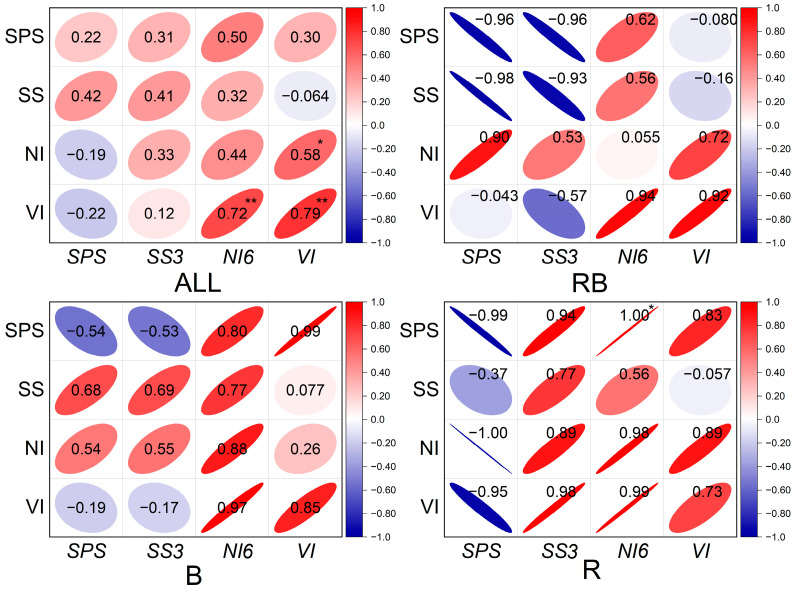
Correlation coefficient analysis between activities of enzymes and related gene expression levels. SPS, sucrose phosphate synthase; SS, Sucrose synthase; NI, neutral invertase; VI, vacuolar acid invertase. *SPS*, sucrose phosphate synthase gene; *SS3*, Sucrose synthase gene; *NI6*, neutral invertase gene; *VI*, vacuolar acid invertase gene. RB, R, and B represent combined red and blue light treatments, monochromatic red light treatments, and monochromatic blue light treatments, respectively. ALL represent four treatments, which include white light treatment, RB, R, and B. Double asterisks indicate a significant correlation at *p* < 0.01. Single asterisks indicate a significant correlation at *p* < 0.05.

The correlation analysis showed that the activities of sucrose-metabolizing enzymes were not always consistent with the expression of their related genes. The SPS activity was negatively correlated with *SPS* expression level under R, B, and RB treatment. With regard to SS, its activity positively correlated with its related gene expression under R and B treatments but negatively correlated with its related gene expression under RB. In contrast, the activities of NI and VI maintained a positive correlation with the expression of *NI6* and *VI* (Figure 5).

## 3. Discussion

With the wide application of LED in photobiology research, it provides great advantages and convenience in revealing the effects of monochromatic light on plant growth and development, especially photo-morphogenesis [3]. Here, our study demonstrated the different regulation patterns of red and blue light on the accumulation and translocation of photosynthetic products in tomato seedlings using different LED light spectra.

For plants, biomass accumulation depends on the P_n_ and leaf areas [24]. In this study, the highest biomass obtained under RB was attributed to RB-induced increases in leaf area (Table 1) and P_n_ (Figure 1A). However, compared with white light control, the decreases in biomass of monochromatic red and blue light could be explained by the significant decline in the leaf areas (Table 1). Similar results were also reported by Izzo, et al. [25]. Compared with the other three treatments, R led to a significant decrease in g_s_ and in turn caused a marked decline in Ci (Figure 1B,C), which normally leads to a decrease in P_n_ [26]. Nevertheless, the P_n_ did not differ significantly among R, B, and W (Figure 1A). This phenomenon might lie in the fact that the red-light-induced increase in g_m_ could counteract the side effect of the Ci decrease on P_n_ because g_m_ is another crucial physiological factor influencing leaf P_n_. An increase in g_m_ could promote intercellular CO_2_, transferring the sub-stomatal cavity into the chloroplast up to the carboxylation site [27,28], thereby improving plant photosynthetic performance [29].

In this study, Ci was significantly decreased under monochromatic red light compared with other treatments (Figure 1), but the δ^13^C levels under monochromatic red light were the largest in the leaves, stems, and roots (Table 3). This is consistent with previous research of Castillo-Argaez, et al. [30], who found that there was a negative linear correlation between δ^13^C values and Ci. Furthermore, adding red light alleviated the blue-light-caused decreases in δ^13^C (Table 3), which further demonstrates the light-quality-dependent photosynthetic carbon fixation. In our present study, red light promoted the transportation and allocation of photosynthetic products from leaves to roots in tomatoes, as shown by the significantly higher ^13^C distribution of roots and lower ^13^C distribution of leaves under R and RB than those under W and B (Figure 2). These results indicated that red light not only could promote photosynthetic carbon fixation [31,32] but also facilitate the long distance of photosynthetic product transportation from leaves to other organs. However, the specific mechanism still needs to be further investigated.

Sucrose is a major photosynthetic product and the principal form in which carbon is transported from mature source leaves to other sink organs, thereby maintaining heterotrophic metabolism and growth or for storage as sucrose or starch [33,34]. In plants, the synthesis, accumulation, and transportation of sucrose depend on the concerted action of sucrose-metabolizing enzymes, including SPS, SS, VI, and NI [12]. The activities of SPS, SS, NI, and VI play vital roles in contributing to the sink strength of plants [34,35]. Our results presented here indicated that monochromic red and a combination of red and blue light could regulate the plant morphology and photosynthesis by the effects on the metabolism of carbohydrates into glucose, sucrose, and starch, mainly through the enhanced activities of SPS, SS, NI, and VI (Figure 3). It is well-known that SPS is the main enzyme in sucrose synthesis [36], while VI and NI are two important enzymes in the degradation of sucrose [37,38]. SS is the only sucrose-metabolizing enzyme that can catalyze both the synthesis of sucrose from fructose and UDP-glucose and the cleavage of sucrose to fructose and UDP-glucose [13]. However, the optimal sucrose synthesis and sucrose degradation activity of SS are pH-dependent. In tomatoes, SS was mainly involved in catalyzing the synthesis of glucose and fructose to sucrose [13]. Increasing SPS activity promotes sucrose formation in plant leaves, while the increased activity of SS could cleave sucrose induced to biosynthesis via producing uridine diphosphate glucose. In the present study, red light was demonstrated to be more efficient in enhancing the activity of SPS and SS, while blue light could effectively enhance the activity of VI and NI, respectively (Figure 3), A similar study reported that sucrose metabolism in tomato seedling leaves affected light conditions via enhancing the activity of SS and SPS [8]. Therefore, the increased sucrose, glucose, and starch in tomato leaves under RB and R has mainly resulted from increased activities of sucrose-metabolizing enzymes, especially SPS and SS (Figure 3).

The transcript levels of these four genes were investigated by a few researchers as affected by different light spectra. These studies mainly focused on one or two enzymes involved in sucrose synthesis or metabolism and related gene responses to different light conditions [25,39,40]. In our study, we intended to reveal the potential relationship between the activities of sucrose-metabolizing enzymes and the light-quality-regulated related gene expression. In plants, the sucrose-metabolizing enzymes are encoded by multiple genes [16,18]. Most importantly, the expression of these genes exhibits significant spatiotemporal variability and differences in response to environmental changes [41]. For instance, among those identified NI genes, *NI6* was proven expressed in all organ tissues and played important roles in the regulation of sucrose metabolism [18]. Thus, we chose four widely investigated genes that encoded sucrose-metabolizing enzymes, including *SPS*, *SS3*, *NI6*, and *VI*. Compared with W, the transcriptions of *SPS*, *SS3*, *NI6*, and *VI* were upregulated in tomato leaves exposed to monochromatic red, blue, and a combination of red and blue light, as shown by the significantly higher gene expression levels under R, B, and RB (Figure 3A–L). In the present study, the relative gene expression of these investigated genes, especially *SPS* and *SS3,* was not in line with the change tendencies of their corresponding enzyme activities under different light treatments (Figure 2 and Figure 3). For instance, under R and B treatments, only the SPS enzyme was negatively correlated with *SPS* gene expression levels. However, both SPS and SS enzymes were negatively correlated with *SPS* and *SS* gene expression levels, respectively, under RB (Figure 5). There might be three reasons for this phenomenon: first, post-mRNA regulation of transcription and various post-translational modifications might result in enzyme activity variety [42]; second, the mRNA transcript levels were not completely consistent with the protein levels, and there may be time differences [43]; third, other genes in the gene family might be involved in the enzyme activity of light quality regulation. Therefore, ongoing research is still needed from the multi-omics level, including single-cell transcriptome and spatiotemporal omics.

## 4. Materials and Methods

### 4.1. Materials and Growth Conditions

Tomato seeds were sown in plastic trays filled with peat–vermiculite (3:1, V/V) and germinated in an environmentally controlled growth chamber (Nanjing Hengyu Instrument Equipment Manufacturing Co., Ltd., Nanjing, China). The sun-like white (W, 380–780 nm) LEDs were used as the light source with light intensity and photoperiod at 150 μmol m^−2^ s^−1^ and 12 h (tomatoes are day-neutral plants, and we use the photoperiod of 12 h to exclude the effect of light duration on tomato plant growth), respectively. The day/night temperature, CO_2_ level, and air relative humidity were set at 26 ± 2 °C/18 ± 2 °C, 400 μmol mol^−1^, and 65%, respectively. The CO_2_ levels, day/night temperature, and air relative humidity in the growth chamber were automatically controlled using carbon dioxide concentration sensors (RS-CO2-NO1-2, JD Renke, Shandong, China) and temperature and humidity sensors (SHT31, Sensirion, Stafa, Switzerland). Every other day, the light intensity of each light treatment was monitored using a light intensity sensor using a light meter (LI-250A, LI-COR, USA) and maintained the photosynthetic photon flux density (PPFD) at 150 μmol m^−2^ s^−1^. At 30 days after sowing, the similar-size and healthy tomato seedlings with three true leaves were transplanted into plastic pots (0.7 L, one plant per pot) with a mixture of peat, vermiculite, and vermiculite (3:1:1, *v*/*v*/*v*) substrate and grown in the same growth chamber for another 7 days before undergoing light treatment.

### 4.2. Light Treatment

After these plants acclimated to the growth condition, these plants were randomly treated with different light treatments. There were four light treatments: monochromatic red (660 nm) LED light (R), monochromatic blue (450 nm) LED light (B), and combined red and blue LED light (RB, R:B = 1:1). These plants exposed to sun-like white LED light (W) were used as control. The light intensity at the top of plant canopies for all the treatments was measured using a light meter (LI-250A, LI-COR, USA) and maintained the PPFD at 300 μmol m^−2^ s^−1^. The total light intensity of all the light spectra for different light treatments was measured using a spectroradiometer (AvaSpec-ULS2048x64-EVO, Avantes, Apeldoorn, The Netherlands). The details of light treatments were summarized in Table A1. Throughout this study, other environmental factors were maintained at similar levels to those at the seedling stage. These plants in different light treatments were watered with full-strength Hoagland solution every 4 days. This study was independently carried out three times.

### 4.3. Gas Exchange Measurement and Sample Collection

At 14 days of light treatment, the second-youngest fully expanded leaves from different light treatments were used for gas exchange as in the method of Bian, et al. [44] using a portable photosynthetic system (LI-6800F, Li-Cor Biosciences, Lincoln, NE, USA). During the measurement, the temperature, light intensity, and CO_2_ concentration in the leaf chamber of the LI-6800F were controlled at 25 °C, 300 μmol m^–2^ s^–1^, and 400 μmol mol^–1^, respectively. The actinic light was provided by the red/blue light sources of the leaf chamber. Apparent mesophyll conductance (g_m_) was calculated as P_n_/C_i_ [45], where C_i_ is intercellular CO_2_ concentration.

The roots, stems, and the second-youngest fully expanded leaves were immediately collected and treated with liquid nitrogen after gas exchange measurement, which were used for total RNA extraction and the determination of carbohydrate contents and the activities of carbohydrate metabolism enzymes.

### 4.4. Biomass and Plant Growth Determination

The plants of different light treatments were collected after being treated for 28 days. The roots, stems, and leaves of these plants were separated for fresh weight (FW) measurement and then dried in an oven at 85 °C for 72 h for the determination of dry weight (DW).

### 4.5. Carbohydrate Metabolism Enzyme Activity Measurement

The enzyme extraction was carried out as in the method of Li, et al. [8] with slight modification. Frozen plant tissue (2.0 g) was grounded using an ice-cold buffer containing 50 mM 4-(2-hydroxyethyl)-1-piperazineethanesulfonic acid (HEPES)/NaOH (pH 7.5), 0.5% (*w*/*v*) of polyvinylpyrrolidone (PVP), 0.05% (*v*/*v*) TritonX-100, 10 mM MgCl_2_, 1 mM EDTA, 0.1% (*w*/*v*) bovine serum albumin (BSA), and 2.5 mM dithiothreitol (DTT). The extracts were centrifuged at 12,000× *g* for 20 min at 4 °C. The supernatant was collected and used as the crude enzyme for the determination of VI, NI, SS, and SPS enzyme activities.

The SS activity was assayed by measuring the absorbance at 480 nm [46]. The reaction mixture contained 50 μL crude enzyme, 10 μL of distilled water, 15 μL of 100 mM uridine diphosphate glucose (UDPG), 20 μL of 50 Mm MgCl_2_, 50 μL of 50 mM HEPES-NaOH (pH 7.5), and 15 μL of 100 mM fructose. The reaction mixture was incubated at 30 °C for 30 min and then added 200 μL of 2 M NaOH and 1.5 mL concentrated hydrochloric acid. The mixture was diluted with 0.5 mL 0.1% hydroquinone and incubated at 30 °C for another 10 min before absorbance measurement. The measurement of SPS activity was similar to the determination of SS activity by replacing 100 mM fructose with 100 mM fructose 6-phosphate (Fru6P) [47].

The activities of VI and NI were measured photospectrometrically according to Jiang, et al. [48]. The extraction mixture contained 50 μL reaction buffer (0.1 M acetic acid buffer and 1% sucrose, pH 5.5) and 50 μL crude enzyme. The mixtures were incubated at 34 °C for 1 h, while 50 μL crude enzymes with 50 μL distilled water incubated at 100 °C for 10 min were used as control. To stop the reaction, we added 1.5 mL of 3,5-dinitrosalicylic acid and then boiled at 100 °C for 5 min. The mixture was diluted to 25 mL with distilled water. The absorbance monitored at 540 nm was used for calculating VI activity. The determination of NI activity was similar to the assay of VI except that the reaction was performed in phosphate buffer (pH 7.5).

### 4.6. Carbohydrate Content Measurement

The method of Buysse and Merckx [49] was used for the measurements of glucose, sucrose, and starch contents. Briefly, freeze-dried plant samples (0.20 g) were extracted in 25 mL 80% ethanol (*v*/*v*) overnight and the supernatants were analyzed for glucose and sucrose contents. The pellets were collected and boiled for 3 h in 10 mL 2% HCl (*v*/*v*). The supernatants were used for starch content analysis. The glucose, sucrose, and starch absorbance were measured at 540 nm, 540 nm, and 660 nm by a multimode microplate reader (Spark, TECAN, Männedorf, Switzerland), respectively.

### 4.7. Pulse-Labeling Experiments and Analysis of Stable Carbon Isotope Composition

After being treated with different light treatments for 7 days, four plants of each light treatment were randomly selected to carry out a pulse-labeling experiment in the same growth chamber as described by Pump and Conrad [50].^13^CO_2_ (99 atom % ^13^C, Shanghai Chemical Research Institute, Shanghai, China) was injected into the pulse-labeling system (one tomato plant per system) with an interval at 1.5 h. The percentage of ^13^CO_2_ and CO_2_ concentration in the pulse-labeling system was kept at 1% and 400 μmol mol^–1^, respectively.

On the third day after the injection of ^13^CO_2_, these tomato plants were destructively sampled. The total organic carbon and the stable carbon (^13^C/^12^C) isotope ratios in the leaves, stems, and roots of plants were determined as in the method by Pump and Conrad [50] and Serret, et al. [51]. The rations of ^13^C/^12^C were expressed as δ^13^C, respectively, and calculated as

(1) δC(‰)13=C13/C12sampleC13/C12standard−1×1000‰

where “sample” and “standard” refer to the plant material to international secondary standards of known ^13^C/^12^C ratio, respectively. Based on the measurements of laboratory standards, the analytical precision was 0.10‰ for δ^13^C (‰).

The distribution of ^13^C_i_ in different organs of plants was calculated as

(2) C13i%=C13C13+C12×Ctotal

where C_total_ refers to the total organic carbon in different organs of plants. Dried samples were ground with a ball mill (MM2000) and analyzed for total organic carbon by an elemental analyzer (Vario PYRO cube, Elementar Analysensysteme GmbH, Langenselbold, Germany).

### 4.8. Total RNA Extraction and qPCR Analysis

At 14 days of light treatment, the samples of roots, stems, and the second-youngest fully expanded leaves were collected. The total RNA of plant tissues was isolated using the RNeasy Plant Mini RNA isolation kit (Qiagen, Hilden, Germany) according to the manufacturer’s instructions. The total RNA was treated with RNase-free DNase I (Invitrogen, Carlsbad, CA, USA) to prevent any genomic DNA contamination and then reverse-transcribed using RNase-free DNase I (Sigma-Aldrich, Poole, UK) as in the method of Bian, et al. [6]. The cDNA fragments were used as templates to quantify the expression of target genes using a CFX Connect™ Real-Time PCR Detection System (Bio-Rad, Hercules, CA, USA). The qPCR was performed with SsoFast™ EvaGreen^®^ Supermix (Bio-Rad) and gene-specific primer mix at 0.2 μM in the volume of 20 μL. The following program was used for PCR amplification: 95 °C for 30 s, followed by 40 cycles at 95 °C for 5 s, 60 °C for 5 s, and a melt curve (65–95 °C). The primers described by Sangu, et al. [17] were used to quantify the expression of *SPS* (Gene ID: AB051216), sucrose synthase gene *SS3* (Gene ID: AJ011319), while the primers reported by Zhang, et al. [41] and Coluccio Leskow, et al. [18] were vacuolar invertase gene *VI* (Gene ID: AF465612) and neutral invertase gene *NI6* (Gene ID: 100134879), respectively. The primer information used in this study was summarized in Table A2.

### 4.9. Data Analysis

The data of this study were analyzed by one-way ANOVA using SAS 8.1 software (SAS Institute, Inc., Cary, NC, USA). The significant differences among treatments were tested by Duncan’s multiple range test (*p* < 0.05). The data were presented as means ± SEs.

## 5. Conclusions

Our study used isotope tracing technology for the first time to systematically analyze different red and blue light quality-regulated photosynthetic products from leaves to other organs of tomato plants. Red light promoted the long-term transfer of fixed carbon from leaves to roots, while blue light was more effective in promoting fixed carbon transfer from leaves to stems. We also found that a combination of red and blue light has a complementary gain effect on fixed carbon transfer and regulating sucrose metabolism enzyme activities and related gene expression. This phenomenon might be attributed to the complexity regulation of the light quality on synthesis and activity of SPS, SS, NI, and VI from transcriptional and post-transcriptional levels. To reveal the underlying mechanism of light spectra on the metabolism and translocation of photosynthetic products in plants, further studies are still needed. In the future, the data of single-cell transcriptome and spatiotemporal omics may shed a green light on revealing the molecular mechanism of light spectra on biosynthesis and the translocation of photosynthetic products in plants.

## Figures and Tables

**Table 1:** The effects of light spectra on plant growth of tomato seedlings.

	Leaf Area (cm^−2^)	Dry Weight (DW, g)	Biomass Distribution Ratio					
			Leaves		Stems		Roots	
W	325.62 ± 46.89 b	1.11 ± 0.10 b	53.31 ± 0.80% a		37.09 ± 0.64% d		9.60 ± 0.51% b	
RB	476.09 ± 53.4 1 a	1.79 ± 0.26 a	43.78 ± 2.1% c	**−9.53%**	44.01 ± 1.1% a	**6.92%**	12.21 ± 0.53% a	**2.61%**
R	266.01 ± 38.25 bc	0.93 ± 0.04 c	47.74 ± 2.6% b	**−7.57%**	40.80 ± 0.40% c	**3.71%**	11.46 ± 1.2% ab	**1.86%**
B	205.86 ± 20.17 c	0.87 ± 0.08 c	48.30 ± 1.1% b	**−5.01%**	41.96 ± 0.61% b	**4.87%**	9.74 ± 1.3% b	**0.1%**

Note: the bold data in columns 5, 7, and 9 of this table represent the difference in the biomass distribution ratio between the treatments and the white light control. W, RB, R, and B represent white light treatments, combined red and blue light treatments, monochromatic red light treatments, and monochromatic blue light treatments, respectively. DW, dry weight. Data represent the mean ± SE (n = 6–9).

**Table 2:** Effects of light spectra on carbohydrate contents in tomato seedlings.

	W	RB	R	B
Leaves (mg g^−1^)				
Glucose	7.33 ± 0.22 d	18.69 ± 0.35 a	10.62 ± 0.30 b	8.35 ± 0.41 c
Sucrose	6.40 ± 0.10 c	8.34 ± 0.31 a	7.64 ± 0.32 b	4.88 ± 0.26 d
Starch	5.08 ± 0.16 b	8.40 ± 0.09 a	5.34 ± 0.20 b	4.56 ± 0.23 c
Stems (mg g^−1^)				
Glucose	11.34 ± 0.24 b	20.86 ± 0.55 a	11.19 ± 0.28 b	8.05 ± 0.31 c
Sucrose	1.70 ± 0.20 c	3.31 ± 0.07 a	2.03 ± 0.01 b	1.71 ± 0.09 c
Starch	4.12 ± 0.04 b	6.88 ± 0.28 a	3.33 ± 0.05 d	3.69 ± 0.02 c
Roots (mg g^−1^)				
Glucose	8.69 ± 0.23 b	9.50 ± 0.36 a	8.61 ± 0.35 b	8.91 ± 0.03 b
Sucrose	5.26 ± 0.11 a	3.44 ± 0.11 b	3.20 ± 0.07 b	2.18 ± 0.09 d
Starch	4.33 ± 0.09 a	3.13 ± 0.94 bc	3.55 ± 0.05 b	3.03 ± 0.06 c

Note: data represent the mean ± SE (n = 6–9). The different letters in the same row indicate significant differences among treatments at *p* < 0.05 according to Duncan’s multiple range test. W, RB, R, and B represent white light treatments, combined red and blue light treatments, monochromatic red light treatments, and monochromatic blue light treatments, respectively.

**Table 3:** Effects of light spectra on organic carbon content and isotope composition of carbon in tomato plants.

Treatments	Organic Carbon (g g^−1^)			δ^13^C (‰)		
	Leaves	Stems	Roots	Leaves	Stems	Roots
W	0.72 ± 0.04 a	0.59 ± 0.05 c	0.62 ± 0.02 b	−41.61 ± 0.78 a	−42.43 ± 1.21 d	−31.99 ± 0.78 b
RB	0.69 ± 0.07 a	0.66 ± 0.02 a	0.80 ± 0.04 a	−41.64 ±1.53 a	−31.14 ± 0.91 b	−36.50 ± 0.45 c
R	0.79 ± 0.08 a	0.64 ± 0.07 b	0.69 ± 0.05 b	−41.27 ± 0.54 a	−27.86 ±1.08 a	−16.93 ± 0.19 a
B	0.67 ± 0.06 a	0.61 ± 0.05 b	0.71 ± 0.07 b	−49.53 ± 0.24 b	−35.50 ± 2.13 c	−45.53 ± 0.31 d

Note: data represent the mean ± SE (n = 6–9). The different letters in the same row indicate significant differences among treatments at *p* < 0.05 according to Duncan’s multiple range test. W, RB, R, and B represent white light treatments, combined red and blue light treatments, monochromatic red light treatments, and monochromatic blue light treatments, respectively.

## Data Availability

The data that support the findings of this study are available from the corresponding author upon reasonable request.

## Appendix A

**Table A1:** The light spectral details of light sources used in this study.

	B	R	RB	W
UV-A light (380–400 nm)	0	0	0	5.47 ± 0.32
Blue light (401–500 nm)	300 ± 8.79	0	150 ± 4.16	65.42 ± 3.34
Green light (501–600 nm)	0	0	0	110.82 ± 4.17
Red light (601–700 nm)	0	300 ± 10.42	150 ± 6.31	127.76 ± 6.45
Far-red light (701–780 nm)	0	0	0	5.29 ± 0.27
PPFD (400–700 nm)	300 ± 8.79	300 ± 10.42	300 ± 11.23	300 ± 13.10
Total light intensity	300 ± 8.79	300 ± 10.42	300 ± 11.23	314.76 ± 14.54

**Table A2:** The details of primers used in this study.

Gene Name	Gene ID:	Sequences
*SPS*	AB051216	Forward: GCTTGGACGGGATTCTGATA
		Reverse: TCTCCCCCATCTCACTCATC
*SS3*	LOC543961	Forward: GGATTGAAAGCCACGGAAAAGG
		Reverse: ACCAGGCCTCAAACGAATAGCA
*VI*	AF465612	Forward: TCCGCCTCTCGTTACACATTACTC
		Reverse: CGGTGACTGGTTGTTGAGGATAGG
*NI6*	100134879	Forward: GGAAGCTGCTCCATCCTTGT
		Reverse: GACGTGGCGGTGAATGAGT
